# Dr. Townsend’s Address before the American Society of Dental Surgeons

**Published:** 1855-07

**Authors:** 


### Dr. TOWNSEND'S ADDRESS BEFORE THE AMERICAN SOCIETY OF DENTAL SURGEONS.

*May 8th,* 1855.

It is now fifteen years since the American Society of Dental Surgeons was organized, yet this is the first time that its members have assembled west of the Allegheny mountains.

The Atlantic slope has been heretofore most happy to extend its professional hospitality to the Mississippi Valley, and now at last it has the pleasure of enjoying the exchange of brotherly courtesies.

The reasons for this reciprocity have existed and have been felt and acknowledged long before the inconvenience of compliance could be well overcome, but I think, I may speak with the clearest assurance for my brethren of the East, that they have found a new happiness in thus meeting, and greeting their friends of the West in their own home. They come to you in a spirit that matches fully the welcome you give them. I cannot in justice say less, and I cannot possibly say more for the sentiment with which the brotherhood of the Atlantic this day greets that of the Mississippi.

But there is some thing more than equality of courtesy in exchange; there is a clear claim of right in it.

The dentistry of the West has honorably earned such fair recognition of its claims. The Star of Science, like the Star of empire, westward holds its way, and the center of influence is rapidly travelling toward the center of territory.

I will not undertake to settle the west longitude of Cincinnati from the site of the professional observatory, but, however far it was behind time in the morning of our day, it seems to me that its meridian has rolled itself fairly into the noontide, and that the whole difference of time is lost in the equality of light in which it now stands. I see nothing of the shadow of the Allegheny upon the science of the great valley, nor do the rays of your sun seem to slant any more than they do "down east."

But you are doubtless comfortable enough in the consciousness of your position without the help of our concessions, and it is only because you are justly entitled to a frank acknowledgment that we make it. You are none the richer that you receive your due, but we would be thus much the poorer if we withheld it.

The distance of time and place which we, who were earliest in this movement, realize to-day by its contrast with that beginning of professional association, presses upon us the feeling of many another change which we cannot wholly silence until it has had some sort of utterance. The time, the place, the circumstances are apt for a brief retrospect, and they are full of suggestiveness for the purposes of the present assemblage.

Fifteen years ago there were not more that 1200 practitioners of dentistry in the Union. The United States census for 1850 reports 2923, and the five years since elapsed have added perhaps 200 annually—bringing the number up to 4000. One dentist for every six thousand of the population of the Union. In 1840 there was but one dentist for every 15,000 of the inhabitants. But this numerical increase of three to one must be multiplied three times at least, fairly to render the amount of the demand for the services of the profession. By this estimate dentistry has risen at least nine fold in public and popular requirement and consideration during the little life time of this society.

This growth in mere bulk and weight indicates a lusty youth, and promises magnificent proportions for that full manhood of the profession on which it is rapidly entering. But what shall we say of its scientific and artistic development during this period?

The men who best knew it then and now, would most feel the difficulty of adequately answering the question. The changes are in fact too great to be measured and reported in all their fulness and value.

Contemporary history is always difficult and sometimes dangerous. Exactness in statement and justice in judgment are scarcely attainable of things passing with ordinary rapidity through one's personal experience, but this history, has had the rush of revolution added to the speed of natural progress.

If the analogy to a human life might be borrowed, we would be justified in saying that our profession 15 years ago had just worried through its infancy. Since then its growth in all things has been that upon which the boy enters at the period of his puberty, a period of manhood in all its faculties and functions, wanting only the discipline of time and experience to strengthen his energies. At the beginning of this period there came into existence the first dental college in this country and the world—now we have two others, the Ohio and Philadelphia Colleges.

Our periodicals also fall within this date, and one of them has 2500 subscribers. Facts like these are exceedingly brief in their statement, but they index volumes of progress, and register incalculable achievements. To my mind, however, there is no more striking indication of advancement than in the altered tone of the journalism and societary addresses which a comparison of the two periods discover. Then, the burden of every appeal was the prevalent charlatanism that infested the profession. Now, the educators, the professional authors, and the orators of our meetings have almost forgotten to confess and deplore the degradation of our calling.

It has passed away to such an extent that we are occupied with that kind of self improvement which implies no stinging professional reproach, but rather proves its healthful soundness. Twenty-five years ago not one in five of the practitioners of our art had attained much practical excellence—the exposure of a general incompetency lay in the mouth of almost every man we met; now, how seldom do we meet the old-time deformities of quackery and ignorance, and how generally good is the work that comes from all quarters of the country under the casual inspection of our most accomplished professors. I need not compare the porcelain and plate work, with the sea horse and blacksmithing of the respective periods which we are speaking of; I need not compare the surgery proper of the mouth and antra which has passed into the hands of the profession from those of the sister branches of Medicine. I need not insist upon the admirable control of irregularities, and the perfection of skill and material with which disease is now curatively treated, and the respectful confidence, with which the popular experience has rewarded us. I need not insist that the medical faculty has recognized our fraternal rank in the general healing art. I need not point to the standard elementary books, which have grown into a library upon our shelves. I comprehend all this progress, and express the compass of all this attainment, by saying that we have reached the high point of respecting ourselves. The very preamble of our constitution in which the necessity "to *give* character and *respectability* to the profession, by establishing a line of distinction between the truly meritorious and skillful, and such as riot in the ill-gotten fruit of unblushing impudence and empiricism," has grown obsolete by its own successes. Even the Constitution of the Mississippi Valley Association founded as lately as 1844 expressed the distressed anxiety to elevate the character and standing of our profession, and make it worthy the confidence of an enlightened public. The brief period of 15 years in the one case and the still briefer period of 10 years in the other have so altered the consciousness of the earnest men of those days that they would scarcely think of admitting such mortifying necessities into the programme of an organization to be set on foot for the profession now.

Happily the great majority of dental practitioners now before the American people have attained an average competency and character, and have acquired courage and confidence enough, to assert for themselves the claims of a liberal profession, and are so well relieved of the taint and suspicion of quackery themselves that they can bear the contact of mediocrity, as well and as safely, as the faculties of law, medicine and divinity sustain their respective proportion of discredit for the same cause.

Even in the matter of provision for the classical education of our pupils we compare with the professions of a century's growth in this country so well, that we may proudly challenge a just comparison. The 40,564 practitioners of medicine sustain 36 colleges, or one school to every 1127 members. 23,939 lawyers have 16 schools, an average of one to 1495 members of the bar. Our three dental colleges bear the intermediate ratio of one to every 1333 practitioners. Thus stands the *preparation* that we have made for the systematic education of our profession on a fairly proportioned level with the highest.

The average of results is of course as yet below the standard before us. The students at the medical schools of 1850 were as one to eight of the practising physicians. The law students, one to forty-five. Ours is perhaps as one to forty, or five times less than the proportion which we should sustain to those of the sister healing art. But our oldest college is of no more than fifteen years standing, the next of ten, and the yongest but three, while collegiate education in general medicine counts to a full century of opportunity for its achievement among us. Give us but ten years more for our trial, and we will outrank them by every test that can determine the pretensions of a profession to the honors of a liberal competition. Ten years will put dentistry at par with medicine in its educational method and success, but without invidiousness we are well warranted in claiming a greater exemption from quackery within our ranks to-day than they are compelled to acknowledge.

The priciples of dentistry are better settled, its practice is better justified, its progress is more certain and rapid now than physic can with any show of truth pretend to.

If it be retorted that we are responsible for so much less; that our entire province covers but one branch of remedial practice, although it in truth involves every branch of medical science, we may answer that not one of the several branches which the parent stock bears has been cultivated into an equal perfection with that one which we have severed from the stem and cultivated with an exclusive care.

But we may fairly claim something more than the faithful and succesful husbandry of our own field of scientific truth; we have developed and demonstrated the method and system by which general medicine is yet to be improved, and our example will in good time compel it into conformity. After our plan of individualizing one distinct branch of remedial art, and making all the others justly subsidiary and subordinate to it, medicine will ere long be divided into surgery, therapeutics and obstetrics, at least. Separate colleges will be assigned to each, with an entire faculty of professors, limited in their teaching and controlled in their drift, by the demands of the particular department which the pupil is destined to practice in after life.

In that day of sounder philosophy in theory and better allotment of functions, Doctor of Dentistry will stand as the pioneer and historic type of the fully instructed Doctor of Physic, Doctor of Surgery, and Doctor of Obstetrics. The memory of our quackery will be forgotten as a fault inevitable to our extreme infancy, and the parent profession will be purged of its own, which in truth is a vice of its constitution, not an accident of its infancy, but ripening with its growth into disease that nothing but such reformation as our policy prescribes can effectually remedy.

Thus already both in achievements actually secured, and in prospects as certainly within our immediate reach, we stand in a position, "looking before and after," that may well justify a generous pride, and inspire a courageous confidence. Our observation, to use a nautical expression, satisfies us that we have been sailing on the high sea of adventure in the right direction, our bearings are all right, our distances show fine speed, and our voyage is all fair sailing before us.

Of one of the instrumentalities availably employed for our advancement, hitherto I have said nothing, I have reserved it because it is that which most especially falls within the functions of the present assemblage, and because it asks a stricter criticism than those other agencies which are working something better for our uses; I mean, the voluntary association of the established practitioners of Dentistry throughout the Union, for such purposes as it can be best made to serve.

The American Society was organized, like the Constitutions of the older States, in the midst of a revolutionary struggle. It had in it the best wisdom, and looked to the worthiest objects, that the light of that day afforded, and the wants then urgent demanded. The roll of its first members is the record of the profession's best and worthiest men. They did their work with pure hearts, fervently, and noble fruits have been harvested of their right hand planting; they have ripened richly for our use, and it is for us to replant them now in a larger field.

The changes that have passed upon the condition of the profession since its institution, in the nature of things, demand correspondent changes in its structure. It bears the name of American, but it is not in any tolerable degree national. It is no longer, as at first, representative of the full force, as well as the best spirit, of the fraternity. A number of names so small as it now embraces, might be enough and more than enough for the highest service in the *cultivation* of any science. In authorship and in professional teaching a score or two of able men might be sufficient for a country and an age, but the purpose of a national society is necessarily the aggregation, or at least the thorough representation, of the entire fraternity, in one organic movement. I venture the assertion that the proper and principal qualification of a National, or State, or County society of professional men engaged in practice, is even more a question of numbers than of standing and talent, provided only that standing and talent are embraced within the number.

The exclusion and exposure of quackery, was the exigency of those days, and numbers was a consideration secondary to all the other objects of association. The sifting process was severely and successfully employed; pledges against modes of practice, held to be irregular and open to great abuses, were exacted; practitioners in good standing were subjected to a formal examination by committees of their equals, as a condition of admission to the society; and, in the ardent zeal of the day for purity of doctrine, even the liberty of opinion, and the right of difference in practice were somewhat abridged. The strifes resulting, bred wars within, as well as without, the camp of the faithful, and the consequence has been a narrowing of the membership in proportion to the rigidness of its discipline.

The society did its work, but is it not also obvious that it has almost lost its capacity for the work that is before it? The Apostles of the New Testament Dispensation think so of the Prophets of the Old, and it is the one lesson which history teaches of most practical value to men, that the men of new times must be allowed to organize the institutions and administer the affairs of their own day.

My motives, I am conscious, need no concealment, my personal position and involvements must acquit me of all suspicion of irreverence and ingratitude to the past, to my own compatriots, and to my fellow laborers. My meaning is, therefore, frankly avowed: speaking for myself, and reflecting the sentiments of a large number of the men whose judgment I profoundly respect, I aim at the regeneration, the rejuvenation of the American society, by such adjustment to the present condition of things as may happily make it as truly broad and national in spirit and action, as it is in name and design. Its position in the profession should be changed from *ex*clusiveness to *in*clusiveness. It should be made comprehensive and representative of the whole professional talent of the nation. It cannot answer the best and broadest of its aims in any other way.

I do not think that a national society of educated men must necessarily be a synod of savans, an aristocracy of talent, a first estate in the realm of science, but, that it bases itself really upon a conceded republican equality of rights and duties; and ought to be democratically representative. Let the profession as it is, liberally embracing all that is fairly professional within it, in fact be fully recognized, and let the common interest take the care of the common weal. Association of superiority with mediocrity can work no injury to the common interest or reputation, but must be productive of improvement every way. The profession abstractly has a character which is no longer endangered by the unworthiness of pretenders. Admission to membership on open and even conditions is no endorsement of the standing of individuals, and works no responsibility of the whole for its parts. The common right of election held by all societies is protective enough of all that concerns the association, and the respectful deference of such an acknowledged equality will secure the support and services of the whole body of the great brotherhood.

No member of this society holds that its certificate is the sole evidence of respectability. He does not govern his own etiquette by any such test, and why should he insist upon it as a condition of association in society, any more than in his daily practice? The whole argument may be made to fall fully within a clear statement of it, which I would put in this form: Is it the business of respectable dentists now to purify the profession, or to improve it—to purge, or, to diet and strengthen it,—to administer alteratives or tonics,—to salivate the convalescent patient, or, to prescribe wholesome nourishment and regenerating exercise?

Let us, my brethren, drop the notion that we have our own respectability to care for. It will care for itself, if it needs any; and let us take up that other idea, that our relations to our brethren now demand a frank devotion to their interests, and the general advancement of our calling. Let us turn reverently to our predecessors and our compatriots of the earlier day, and render them the homage due for a wise, brave, and successful championship of our honored calling, and pledge ourselves to as ardent, as devoted, and as worthy an effort to serve the enlarged interests which we have and hold now by inheritance from them.

The essential aims of our association are well expressed in the preamble to the Constitution: "The objects of this society are to promote union and harmony among *all* respectable and well-informed Dental Surgeons; to advance the science by free communication and interchange of sentiments, either verbal or written, between the members, both in this and other countries." The Mississippi Valley Association in different terms expresses the same intention. "We deem it expedient to form an Association, for purposes of mutual improvement in the science and practice of our profession, to promote the exercise of that gentlemanly courtesy which should characterize members of liberal professions in both social and professional intercourse; believing also that by frequent interchange of opinion and observation in practice, by reporting from time to time cases of interest as they occur in individual practice, we may do much to elevate the character, &c."

The American Society of Physicians and Surgeons, and a long list of associations among the cultivators of liberal learning and arts, assign similar objects for similar organizations. The members of the learned professions who feel the necessity of a forward movement, and the friends of general education, are all using the method of such personal conference, discussion and oral communication, on a clear perception of its special advantages and of its remunerating value in the service of professional interest and general learning.

It is not necessary to detail these advantages, or to dilate upon the utility of organizations among professional adepts; they are too well understood, too well warranted by experience, and they are not disputed. I allude to them only to invoke their proper force in urging the endeavor to make our society answer all the ends of which in the nature of things it is capable; and I submit to the best judgment of its members their duty of adopting whatever measures may seem to them necessary and best adapted to put it abreast of the times.

How shall we make its actual agency coextensive with its avowed objects, is the question; and our duty to the profession requires that we solve this question and resolve upon the expedient. I doubt not that this assemblage is competent to the devising of some proceeding which will early and happily answer the question.

